# Loss of epidermal MCPIP1 is associated with aggressive squamous cell carcinoma

**DOI:** 10.1186/s13046-021-02202-3

**Published:** 2021-12-13

**Authors:** Weronika Szukala, Agata Lichawska-Cieslar, Roza Pietrzycka, Maria Kulecka, Izabela Rumienczyk, Michal Mikula, Iwona Chlebicka, Piotr Konieczny, Katarzyna Dziedzic, Jacek C Szepietowski, Giulia Fontemaggi, Janusz Rys, Jolanta Jura

**Affiliations:** 1 Faculty of Biochemistry, Biophysics and Biotechnology, Department of General Biochemistry, Jagiellonian University, Gronostajowa 7, 30-387 Krakow, Poland; 2 Medical Center for Postgraduate Education, Department of Gastroenterology, Hepatology and Clinical Oncology, Marymoncka 99/103, 01-813 Warsaw, Poland; 3 Maria Skłodowska-Curie National Research Institute of Oncology, Roentgena 5, 02-781 Warsaw, Poland; 4 Department of Dermatology, Venereology and Allergology, Wroclaw Medical University, Chalubinskiego 1, 50-368 Wroclaw, Poland; 5 Oncogenomic and Epigenetic Unit, Regina Elena National Cancer Institute, Via Elio Chianesi 53, 00-144 Rome, Italy; 6 Maria Skłodowska-Curie National Research Institute of Oncology, Garncarska 11, 31-115 Krakow, Poland

**Keywords:** MCPIP1, Regnase-1, SCC, Skin cancer, Keratinocyte

## Abstract

**Background:**

Squamous cell carcinoma (SCC) of the skin is a common form of nonmelanoma skin cancer. Monocyte chemotactic protein 1-induced protein 1 (MCPIP1), also called Regnase-1, is an RNase with anti-inflammatory properties. In normal human skin, its expression is predominantly restricted to the suprabasal epidermis. The main aim of this study was to investigate whether MCPIP1 is involved in the pathogenesis of SCC.

**Methods:**

We analyzed the distribution of MCPIP1 in skin biopsies of patients with actinic keratoses (AKs) and SCCs. To explore the mechanisms by which MCPIP1 may modulate tumorigenesis *in vivo*, we established a mouse model of chemically induced carcinogenesis.

**Results:**

Skin expression of MCPIP1 changed during the transformation of precancerous lesions into cutaneous SCC. MCPIP1 immunoreactivity was high in the thickened area of the AK epidermis but was predominantly restricted to keratin pearls in fully developed SCC lesions. Accelerated development of chemically induced skin tumors was observed in mice with loss of epidermal MCPIP1 (Mcpip1^eKO^). Papillomas that developed in Mcpip1^eKO^ mouse skin were larger and characterized by elevated expression of markers typical of keratinocyte proliferation and tumor angiogenesis. This phenotype was correlated with enhanced expression of IL-6, IL-33 and transforming growth factor-beta (TGF-β). Moreover, our results demonstrated that in keratinocytes, the RNase MCPIP1 is essential for the negative regulation of genes encoding SCC antigens and matrix metallopeptidase 9.

**Conclusions:**

Overall, our results provide a mechanistic understanding of how MCPIP1 contributes to the development of epidermoid carcinoma.

**Supplementary Information:**

The online version contains supplementary material available at 10.1186/s13046-021-02202-3.

## Background

The skin is the largest organ in the human body and provides a physical barrier against the external environment. It harbors several types of immune cells crucial for host defense and tissue homeostasis. Any defects in the immune response of the skin may result in excessive infections, autoimmunity or tumors [1]. Skin cancer is the most frequent type of malignancy, and basal cell carcinoma (BCC) and squamous cell carcinoma (SCC) are the most common subtypes [2, 3]. SCC originates from the upper layer of the epidermis and accounts for approximately 30% of nonmelanoma skin cancers (NMSCs). Actinic keratoses (AKs) are the most typical precursor lesions of SCC [3]. SCCs are generally surgically excised and do not recur, but metastasis is reported in approximately 6% of cases [4]. Several risk factors, including long-term sunlight exposure, immunosuppression and genetic factors, have been associated with an increased likelihood of developing skin carcinogenesis and epidermal dysplasia [3, 5]. The worldwide incidence of NMSCs is increasing; this increase is most commonly associated with exposure to UV radiation and population aging [3, 4].

Monocyte chemotactic protein 1-induced protein 1 (MCPIP1), also called Regnase-1, is an RNase with anti-inflammatory properties [6]. It contains a CCCH-type zinc finger motif, which is required for its interaction with RNA, and a PilT N-terminus (PIN) domain, which confers its enzymatic activity [6–8]. The interaction of MCPIP1 with RNAs is based on the recognition of specific stem-loop structures [9], which leads to destabilization of the mRNA. The targets of MCPIP1 include transcripts encoding proinflammatory cytokines (IL-1β, IL-6, IL-12p40 and IL-2) [6, 7, 10], transcription factors (c-Rel and CEBPβ) [11, 12], signal transducers (Ox40) [11] and MCPIP1 itself [13]. MCPIP1 also degrades hairpin precursors of miRNAs, affecting miRNA biogenesis [14].

The fundamental function of MCPIP1 is to prevent excessive inflammation. Mice deficient in MCPIP1 develop a severe inflammatory response, which leads to splenomegaly, lymphadenopathy, hyperimmunoglobulinemia and death within 12 weeks [6, 15]. Recent studies have indicated that MCPIP1 also regulates processes related to cell differentiation, apoptosis, angiogenesis and cancer metabolism [16–19]. These properties were identified by *in vitro* and *in vivo* studies in carcinoma cell lines and xenograft mouse models. In several types of both epithelial and non-epithelial tumors, including clear cell renal cell carcinoma [20], neuroblastoma [21], melanoma [22] and breast cancer [18], the level of MCPIP1 is decreased, suggesting its tumor-suppressive properties (reviewed in [23]).

In the skin, MCPIP1 is expressed mostly in the suprabasal epidermis [24, 25]. Our recent studies indicated that conditional knockout of MCPIP1 in keratinocytes impairs skin homeostasis, which manifests phenotypically as impairment of skin integrity in aged mice [24]. In addition, MCPIP1 has been shown to be involved in the pathology of psoriatic disease [25–28].

As discussed above, accumulating evidence indicates that MCPIP1 plays a role in the development of cancer. However, there are no data regarding the potential function of MCPIP1 in the development of cutaneous SCC, the second most common malignancy in humans. In this study, we investigated whether the expression of MCPIP1 is related to tumor progression in human SCC and in established mouse model of this disease. In the mouse model, we explored the potential mechanisms by which MCPIP1 may modulate tumorigenesis *in vivo*.

## Materials and methods

### Patient tissue samples

Skin biopsies were obtained from patients in the Department of Dermatology, Venereology and Allergology at Wroclaw Medical University. Most SCC and AK skin biopsies were obtained from the scalp, lower lip, cheek and pinna. The mean age in the SCC group (20 patients) was 76 ± 13 (standard deviation (SD)) years and in the AK group (8 patients) was 72 ± 9 years. Prior to surgical excision, the patients were injected with an anesthetic (2% lidocaine and adrenaline) to diminish pain and suppress bleeding. The study was approved by the Bioethics Committee of Wroclaw Medical University (KB-520/2018).

### Animals

To obtain mice with keratinocyte-specific knockout of the *Zc3h12a* gene encoding Mcpip1, the Cre-loxP system was used as described previously [24]. Mcpip1^loxP/loxP^ mice [29], referred to herein as control mice, were crossed with K14^Cre^ mice [30]. All mice were on a C57BL/6NJ background. Animals were housed under specific pathogen-free (SPF) conditions in accordance with the Guide for the Care and Use of Laboratory Animals (Directive 2010/63/EU of the European Parliament), and animal experiments were carried out under a license from the 2nd Local Institutional Animal Care and Use Committee in Kraków.

### Chemical induction of tumorigenesis

To establish 7,12-dimethylbenz[a]-anthracene (DMBA)/12-*O*-tetradecanoylphorbol-13-acetate (TPA)-induced carcinogenesis, the backs of 6- to 8-week-old control and Mcpip1^eKO^ mice were shaved, and a single dose of DMBA (30 µg in 200 µl of acetone; Sigma-Aldrich, St. Louis, MO, USA) was applied. Then, two weeks later, this treatment was followed by twice-weekly application of TPA (15 µg in 200 µl of acetone; Sigma-Aldrich) for 10 weeks. The tumor number and volume were determined twice weekly during the experimental period. At the end of the experimental period, mice were euthanized and tumor samples were collected.

### Cell culture

The human epidermal carcinoma cell line A431 and the HEK293 cell line were obtained from ATCC (Manassas, VA, USA). Cells were cultured at 37 °C in a 5% CO_2_ atmosphere in either Dulbecco’s modified Eagle’s medium-low glucose (Lonza Group Ltd., Basel, Switzerland) for A431 cells or DMEM-high glucose for HEK293 cells. Both media were supplemented with 10% fetal bovine serum (FBS, Sigma-Aldrich). Cells were seeded in 6/12-well culture plates at 30% confluence. The following day, cells were stimulated with 10 ng/ml transforming growth factor-beta 1 (TGF-β1; Cell Signaling Technology, Danvers, MA, USA) for 24, 48 and 72 h.

### Stable transduction with viral vectors

To stably knock down MCPIP1 expression, lentiviral vectors containing control shRNA and shRNA specific for MCPIP1 were used (Sigma-Aldrich). For overexpression, the doxycycline-dependent TetON system was used (with pLIX vectors) as described previously [19]. In brief, A431 cells were seeded in 6-well culture plates at 30% confluence. Viral vectors were added at a multiplicity of infection (MOI) of 50 in the presence of 6 mg/ml polybrene (Millipore, Billerica, MA, USA). After 24 h, the virus-containing medium was replaced with fresh medium for 24 h, and selection was then conducted in medium containing 1 µg/ml puromycin (InvivoGen) for 10 days to select stably transduced cells. To induce the expression of MCPIP1, 1 µg/ml doxycycline (BioShop, Burlington, Canada) was added to the culture medium. When indicated, cells were treated with 5 ng/ml TPA (Sigma-Aldrich) for 24 h.

### RNA isolation and quantitative reverse transcription PCR (qRT-PCR)

For RNA isolation, tumor samples were frozen in liquid nitrogen immediately after collection and stored at -80 °C until analysis. RNA isolation and qRT-PCR were performed as described previously [24]. RNA from cells was isolated using the Fenozol-chloroform method (A&A Biotechnology, Gdynia, Poland). Real-time PCR was performed using SYBR Green Master Mix (A&A Biotechnology, Gdynia, Poland) for analysis of mouse transcripts and with SYBR Green JumpStart Taq ReadyMix (Sigma-Aldrich) for analysis of human transcripts. The sequences of the primers (Sigma-Aldrich) are listed in Table S1 (Additional file 1).

### Protein isolation and western blot analysis

A431 cells were washed with PBS and lysed in radioimmunoprecipitation assay (RIPA) buffer supplemented with protease and phosphatase inhibitors. Protein concentrations were measured with a bicinchoninic acid assay. Electrophoretic separation and western blot analysis were performed as described previously [24]. The antibodies utilized for western blot analysis are listed in Table S2 (Additional file 2). 

### ELISA

Tumor samples that had been frozen and stored at -80 °C were homogenized in PBS containing 1% Triton X-100 (Bio-Shop) and protease inhibitors. The total protein concentration was assessed using a bicinchoninic acid assay. The levels of IL-33, IL-6 and TGFβ1 were measured in duplicate using mouse DuoSet ELISAs (R&D Systems, Minneapolis, Canada) according to the manufacturer’s protocol.

### Histological and immunochemical staining

For immunohistochemical staining of human skin biopsies, formalin-fixed, paraffin-embedded sections were cut (5 μm) and stained using an EnVision+ System-HRP Labeled Polymer Anti-Rabbit Kit (Dako, Glostrup, Denmark) according to the manufacturer’s protocol. Tumor samples from mice were collected, fixed and subjected to immunofluorescence staining as described previously [28]. Sections were examined using Leica Application Suite X (LAS X) image acquisition software and a Leica DMC5400 fluorescence microscope (Leica Microsystems, Wetzlar, Germany). All figures were prepared using ImageJ (National Institutes of Health, Bethesda, MD) and Adobe Illustrator CC (Adobe Systems, San Jose, CA). The antibodies utilized for staining are listed in Table S2 (Additional file 2).

### RNA sequencing (RNA-seq)

Sequencing libraries were prepared from 50 ng of total RNA by using an Ion AmpliSeq™ Transcriptome Mouse Gene Expression Kit (Thermo Fisher Scientific (Thermo), Waltham, MA, USA). In brief, RNA fragments were reverse transcribed and amplified. Then, cDNA was hybridized and ligated with Ion adaptors. Next, cDNA was purified by a magnetic bead-based method. The molar concentration and size selection (125-300 bp) of each cDNA library was determined using a DNA HS Kit in a Bioanalyzer 2100 system (Agilent). Each library was diluted to ~55 pM before template preparation, and up to six barcoded libraries were mixed in equal volumes and used for automatic template preparation in an Ion Chef (Thermo) instrument using reagents from an Ion PI Hi-Q Chef Kit (Thermo), Ion PI Hi-Q Sequencing 200 Kit and Ion PI Chip. Samples were sequenced in an Ion Proton System (Thermo). Raw reads were processed with TorrentSuite version 5.10 and mapped to the AmpliSeq mouse transcriptome reference. Gene abundances were quantified with htseq-count (HTSeq framework version 0.6). Differential gene expression analysis was performed with the R package DESeq2 version 1.18.1. Sequencing data are available in the European Nucleotide Archive under accession number PRJEB45882.

### Functional analyses of transcriptome data

Functional annotation of differentially expressed genes (DEGs; fold change ≥1.5) was performed using bioinformatic tools available *via* the Database for Annotation, Visualization, and Integrated Discovery (DAVID version 6.8, https://david.ncifcrf.gov/home.jsp). Gene lists were searched using Ensembl gene annotations (ENSEMBL_GENE_ID). Selected genes were classified into function-related gene groups using gene ontology (GO) biological process (BP) terms.

### Dual-luciferase assay

The full-length 3’UTRs of *IL33, matrix metallopeptidase 9 (MMP9)* and *SERPINB3/B4* were amplified using gene-specific primers (Table S1, Additional file 1) and inserted into the pmirGLO dual-luciferase plasmid (FJ376737; Promega, Madison, WI, USA) *via* the NheI and SalI sites. All constructs were verified by sequencing. HEK293 cells were seeded in 48-well plates one day before cotransfection with 282 ng of the pmirGLO-3’UTR or pmirGLO-empty vector and 18 ng of pcDNA3.0, pcDNA3.0-MCPIP1 (encoding human MCPIP1) or pcDNA3.0-MCPIP1-D141N using jetPRIME Reagent (Promega, Madison, USA). Twenty-four hours later, the cells were lysed, and the supernatants were analyzed using a Dual-Luciferase Reporter Assay System (Promega) in a Turner Biosystems 20/20n luminometer (Promega). The luciferase activity in each sample was normalized to that of the corresponding pmirGLO-empty vector.

### Statistical analysis

Statistical analyses, including one-way analysis of variance (ANOVA) and Student’s *t*-test, were performed using GraphPad Prism 7 (GraphPad Software, Inc., La Jolla, CA).

## Results

### The MCPIP1 expression profile is changed in AK and SCC skin lesions

**Fig. 1:**
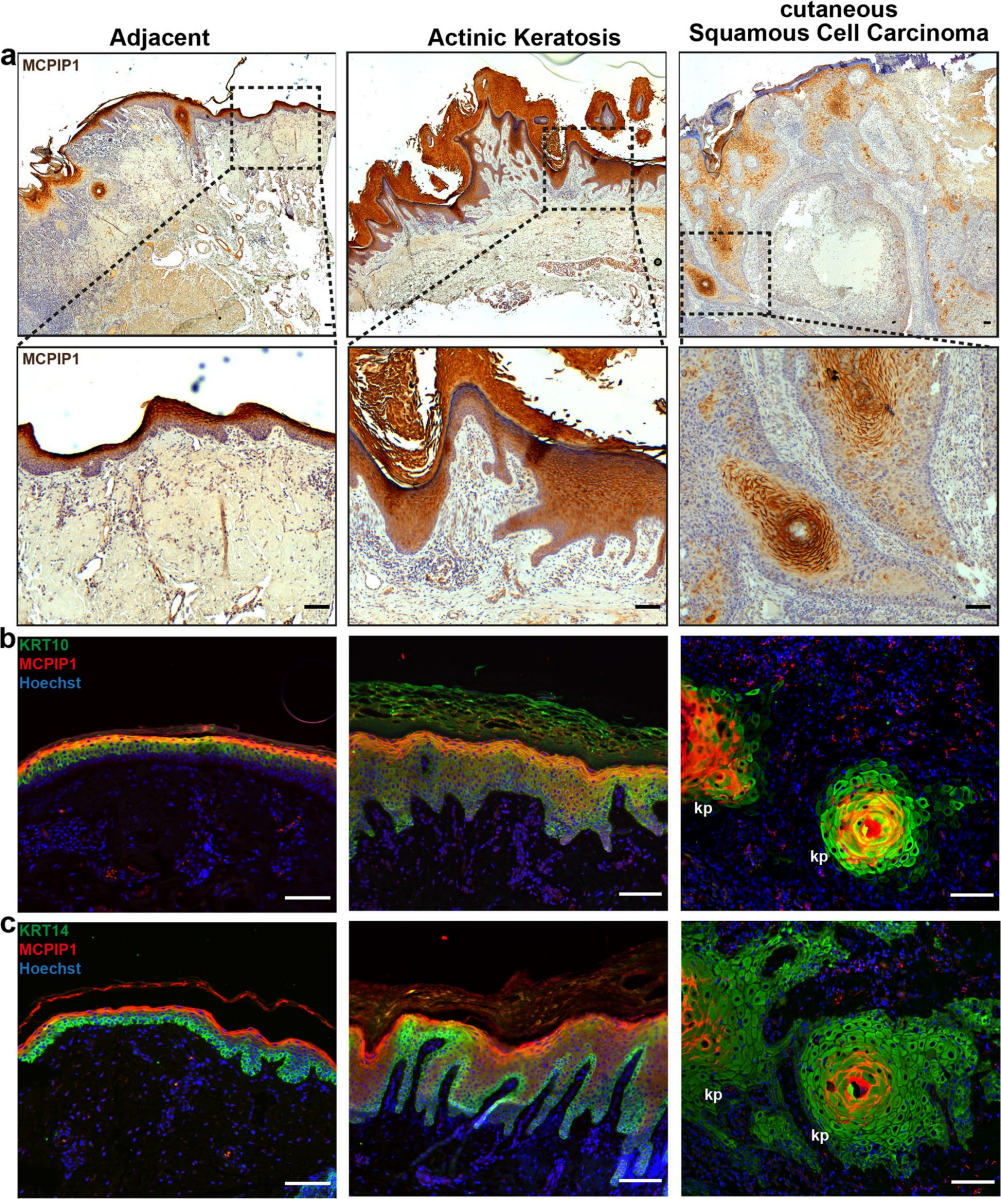
MCPIP1 expression profile in AK and SCC skin lesions.(**a**) Microscopy analysis of MCPIP1 immunohistochemical staining in tumor-adjacent skin, AK skin biopsies and SCC skin biopsies. (**b**) KRT10 and MCPIP1 and (**c**) KRT14 and MCPIP1 immunofluorescence staining of skin sections. kp – keratin pearls. Scale bar: 100 μm

We began our analysis of the potential role of MCPIP1 in SCC pathogenesis by characterizing its distribution in skin biopsies from eight patients with AK and twenty patients with SCC (Fig. 1a). Histological analysis indicated that AK biopsies showed thickening of the epidermis (acanthosis). MCPIP1 immunoreactivity was higher than that in the adjacent skin, particularly in cells that also expressed keratin 10 (KRT10), which is a characteristic for differentiating benign tumors (Fig. 1b). In contrast, in the fully developed SCC biopsies, the overall MCPIP1 expression level was low, and MCPIP1 expression was predominantly restricted to keratin pearls, typical structures observed in cutaneous SCC that are composed of well-differentiated neoplastic squamous cells (Fig. 1a). These cells coexpressed KRT10 and MCPIP1, yet the overall distribution of KRT10 was lost in malignant tissue **(**Fig. 1b and S1a, Additional file 3). KRT14 showed a relatively normal distribution in both adjacent and AK skin samples and was expressed in dividing basal keratinocytes. However, cutaneous SCC samples exhibited enhanced KRT14 immunoreactivity (Fig. 1c and S1b, Additional file 3).

### Accelerated development of chemically induced papillomas in the skin of Mcpip1^eKO^ mice

**Fig. 2:**
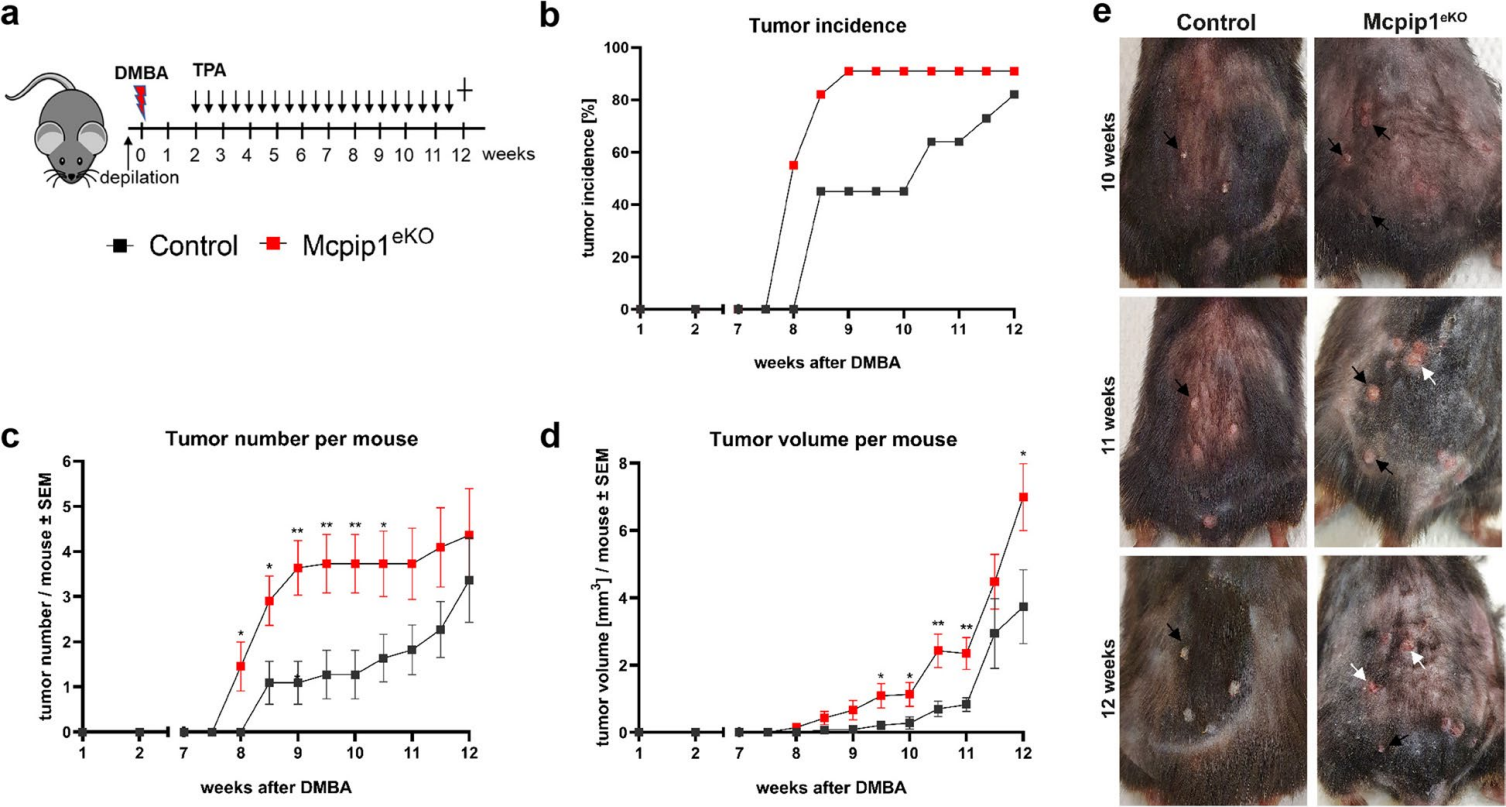
DMBA/TPA-induced epidermal papillomas in Mcpip1^eKO^ mice. (**a**) Schematic diagram showing the timeline of the DMBA/TPA skin carcinogenesis protocol. (**b**) Tumor incidence (percentage of mice with papillomas), *n = 11 mice*. (**c**) Tumor multiplicity (average number of papillomas per mouse). (**d**) Mean tumor volume per mouse. Individual tumor volumes were calculated based on the following equation: volume [mm^3^] = (weight^2^ × height) / 2. The data are shown as the mean ± SEM values; *n = 9-10*. Multiple samples t-test was used to calculate *P-values*. *P < 0.05, **P < 0.01. (**e**) Dorsal view of mice at 10-12 weeks after DMBA treatment. The black arrowheads indicate exophytic papillomas, and the white arrowheads indicate endophytic papillomas

To investigate the potential role of MCPIP1 in the development of skin carcinoma, we utilized a multistage chemical carcinogenesis approach to model the development of SCC in mouse skin with loss of keratinocyte MCPIP1 function (Krt14^Cre^Mcpip1^fl/fl^ mice, herein referred to as Mcpip1^eKO^ mice). Tumors were initiated following one subcarcinogenic dose of the potent carcinogen DMBA. This treatment was followed by twice-weekly application of the tumor-promoting agent TPA [31]. Due to the excessive development of wounds in aging Mcpip1^eKO^ mice, all mice were sacrificed after 12 weeks of DMBA/TPA treatment (Fig. 2a), as recommended by the local ethics committee.

The overall strategy of chemical carcinogenesis was very efficient. After 12 weeks of DMBA/TPA treatment, approximately 86% of the control mice and Mcpip1^eKO^ mice developed papillomas. However, papilloma development was accelerated in case of the Mcpip1^eKO^ mice. In particular, after only 9 weeks, epidermal papillomas had developed in 91% of the Mcpip1^eKO^ mice but in only 45% of the control mice (Fig. 2b). The number and size of tumors increased over time in both types of mice, but more tumors formed in Mcpip1^eKO^ mice (Fig. 2c). Macroscopically, the papillomas that developed in Mcpip1^eKO^ mice were generally larger **(**Fig. 2d**)**, and some infiltrated the dermis (Fig. 2e). In addition, most Mcpip1^eKO^ lesions showed discontinuity of the epidermis, with a scaly, red, ulcerated surface. These features were not observed in the papillomas that developed in the control mice (Fig. 2e).

**Fig. 3:**
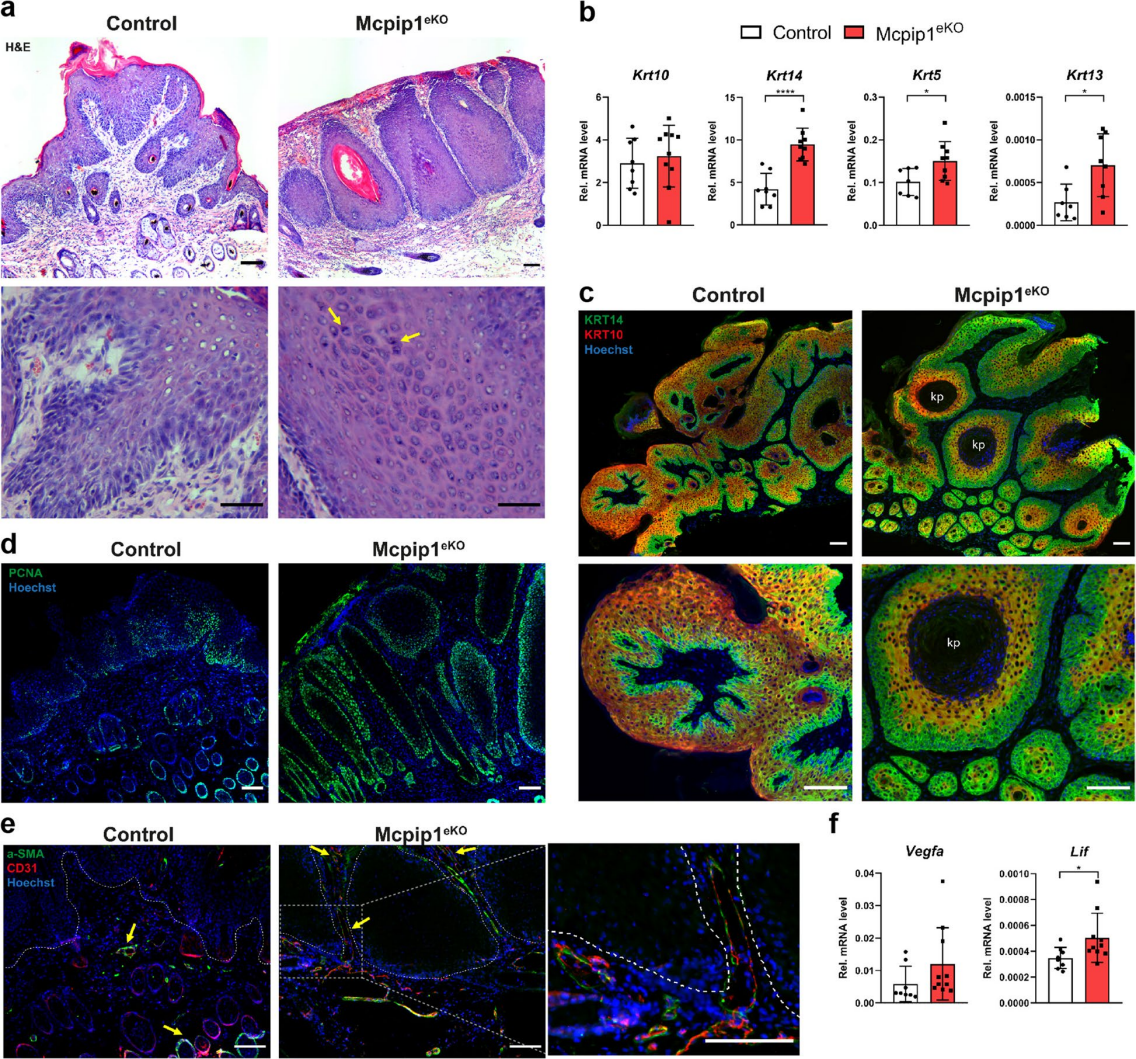
Histological and immunofluorescence analyses of DMBA/TPA-induced Mcpip1^eKO^ papillomas.(**a**) Representative H&E staining of control and Mcpip1^eKO^ papillomas after 12 weeks of DMBA treatment. The yellow arrowheads indicate cells with heterochromatic nuclei. (**b**) qRT-PCR analysis of *Krt10, Krt14, Krt5* and *Krt13* expression. *n = 8-9*. (**c**) KRT10 and KRT14 and (**d**) PCNA immunofluorescence staining in papilloma sections. (**e**) Representative immunofluorescence staining of α-SMA and CD31 in Mcpip1^eKO^ and control papillomas. The arrowheads indicate double-positive cells (indicating vessel-like structures). (**f**) qRT-PCR analysis of *Vegfa* and *Lif* expression. *n = 8-10*. The data are shown as the mean ± SD values. Unpaired t-test was used to calculate *P-values*. *P < 0.05, ****P < 0.0001. kp - keratin pearls. Scale bar: 100 μm

Histological analysis confirmed that in the control mice, the papillomas that formed were exophytic (protruding outward), whereas most Mcpip1^eKO^ mice developed endophytic skin papillomas (Fig. 3a). 80% of the papillomas in the Mcpip1^eKO^ mice showed signs of differentiated SCC. We noted the presence of atypical mitotic cells and infiltration of cells with heterochromatic nuclei into the dermis as well as the formation of typical structures of SCC—keratin pearls. These abnormal morphological changes were only occasionally observed in control mice (less than 30%).

We next analyzed the expression level and distribution of certain keratins, whose expression pattern is often altered during malignant transformation. Mcpip1^eKO^ papillomas were characterized by elevated transcriptional expression of *Krt5, Krt13* and *keratin 14 (Krt14)*, indicating a highly proliferative phenotype (Fig. 3b). Immunohistochemical staining revealed altered translational expression of KRT14, which, under physiological conditions, is expressed in mitotically active basal cells, and of KRT10, the marker of differentiating keratinocytes. Compared with those in the control mice, the papillomas in Mcpip1^eKO^ mice showed increased expression levels of KRT14; in contrast, KRT10 expression was clearly reduced (Fig. 3c). Consistent with this finding, the number of PCNA-positive cells was obviously higher in Mcpip1^eKO^ mice (Fig. 3d).

The Mcpip1^eKO^ tumors were also characterized by accelerated angiogenesis. Immunostaining of CD31 and alpha-smooth muscle actin (α-Sma) revealed that angiogenesis was enhanced in the tumors of Mcpip1^eKO^ mice, in which an intrinsic vascular network already developed around the neoplastic mass (Fig. 3e). This phenomenon was not observed in control mice, which exhibited a relatively normal distribution of skin blood vessels exclusively in the hypodermis (Fig. 3e). Consistent with this finding, the mRNA levels of the proangiogenic factors *vascular endothelial growth factor a (Vegfa)* and *leukemia inhibitory factor (Lif*) were increased in the tumors of Mcpip1^eKO^ mice (Fig. 3f).

### Loss of MCPIP1 function results in extensive transcriptome changes upon DMBA/TPA challenge

**Fig. 4:**
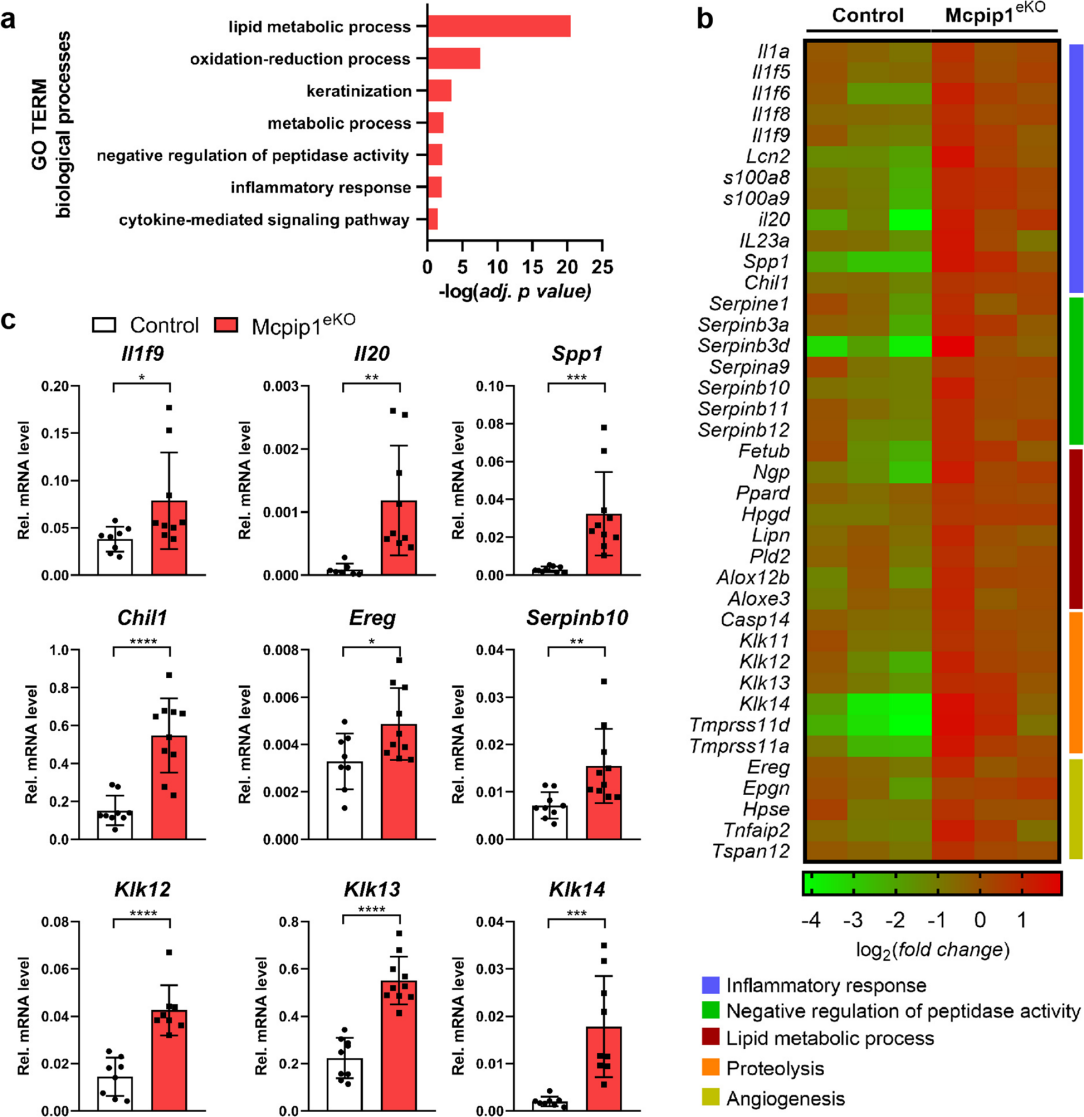
Global transcriptome changes in Mcpip1^eKO^ papillomas. RNA-seq was performed on RNA isolated from three Mcpip1^eKO^ and three control papillomas after 12 weeks of DMBA/TPA challenge. (**a**) GO enrichment analysis of genes with upregulated expression in Mcpip1^eKO^ mice compared to control mice (adj. *P-*value < 0.05 and fold change ≥ 1.5). (**b**) Heatmap showing DEGs selected from GO enrichment analysis. (**c**) qRT-PCR analysis of *Il1f9, Il20, Spp1, Chil1, Ereg, Serpinb10, Klk12, Klk13* and *Klk14* expression. The data are shown as the mean ± SD values; *n = 8-10*. Unpaired t-test was used to calculate *P-values*. *P < 0.05, **P < 0.01, ***P < 0.001, ****P < 0.0001

We next performed RNA-seq on RNA isolated from three Mcpip1^eKO^ mouse and three control mouse papillomas after 12 weeks of DMBA/TPA challenge. A total of 474 and 261 transcripts were significantly up- and downregulated (fold change>1.5 and adj. *P-*value<0.05), respectively. GO analysis revealed that the DEGs with increased expression in Mcpip1^eKO^ papillomas were significantly enriched in several biological processes, including ‘lipid metabolic process’, ‘keratinization’, ‘negative regulation of peptidase activity’, ‘inflammatory response’, and ‘cytokine mediated signaling pathway’ (Fig. 4a). This finding was generally consistent with some already described functions of MCPIP1, such as modulation of inflammatory reactions and lipid metabolism. We also identified transcripts related to angiogenesis and proteolysis among the DEGs significantly upregulated in Mcpip1^eKO^ papillomas; however, the abovementioned categories were not identified as significantly changed in GO analysis (Fig. 4b).

Increased expression of a plethora of inflammation-related genes was observed in Mcpip1^eKO^ papillomas. This set of genes included genes that had already been described to be responsive to MCPIP1 RNase activity, such as *S100a8/a9, Lcn2, Il1a, Il1f5/f6/f8* and *-f9* [24, 25, 27], and several novel genes, in particular *Il20, secreted phosphoprotein 1 (Spp1)* and *chitinase-like 1 (Chil1)* (Fig. 4b and c). Another group of DEGs significantly upregulated in Mcpip1^eKO^ papillomas comprised members of the family of genes encoding serpins (i.e., *Serpinb3a/b3d/b10/b11*), regulators of peptidase activity (Fig. 4b and c). We also shortlisted a group of DEGs related to proteolysis that included several members of the kallikrein-related peptidase family (*Klk11/12/13* and *-14*) and *caspase 14 (Casp14)* (Fig. 4b and c). Finally, we noted enhanced transcriptional expression of some positive modulators of angiogenesis, such as *epiregulin (Ereg), epigen (Epgn), heparanase (Hpse)* and *tumor necrosis factor alpha-induced protein 2 (Tnfaip2)* (Fig. 4b and c).

### Keratinocyte MCPIP1 modulates the expression of genes encoding SCC antigens and MMP9

**Fig. 5:**
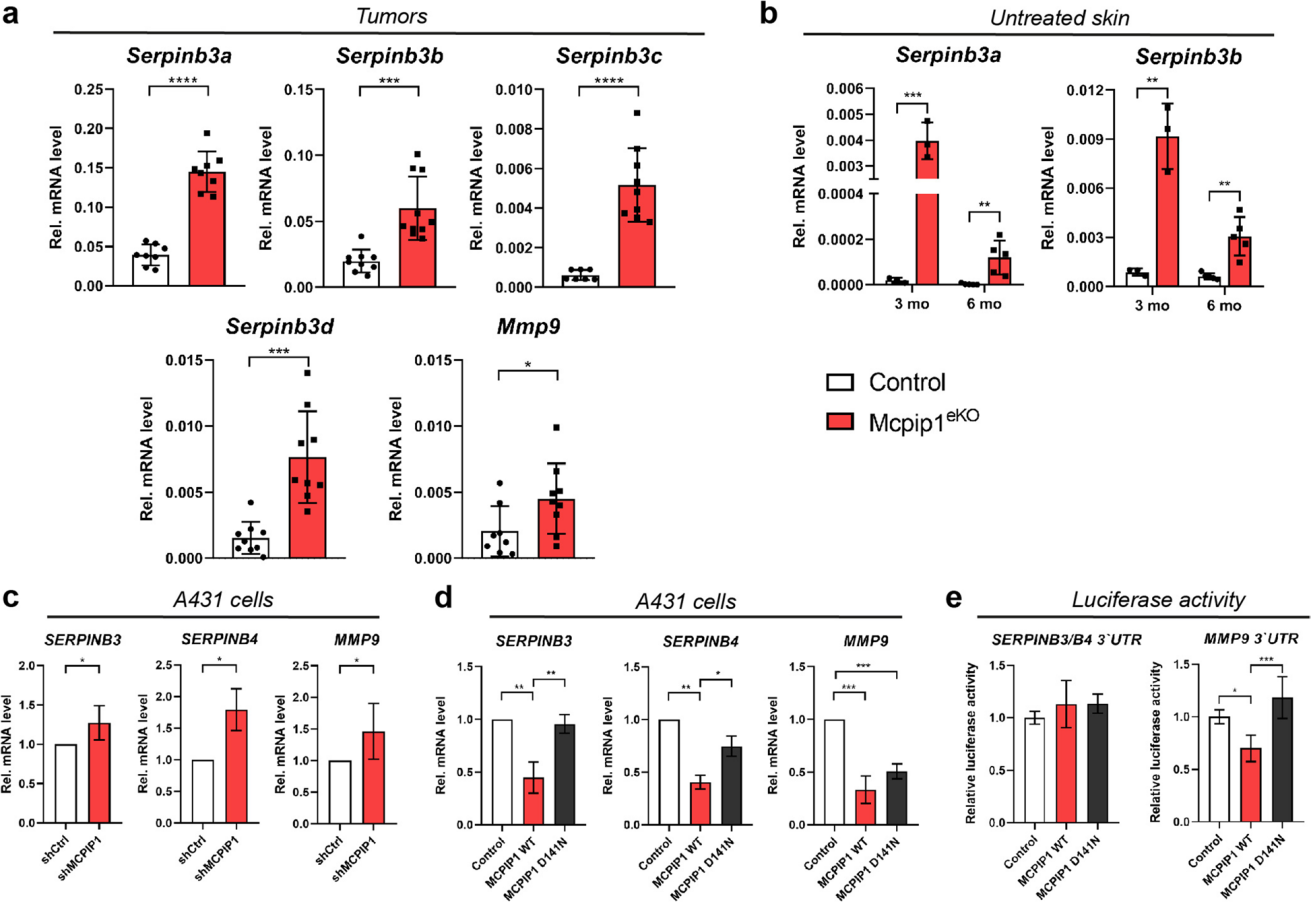
The expression level of MCPIP1 negatively correlates with the expression levels of the genes encoding SERPINB3/B4 and MMP-9. (**a**) qRT-PCR analysis of *Serpinb3a, -3b, -3c, -3d* and *Mmp9* expression in papillomas. *n = 8-10*. (**b**) qRT-PCR analysis of *Serpinb3a* and *-3b* expression levels in whole skin lysates of 3-month-old and 6-month-old mice. *n = 3-5*. (**c**) qRT-PCR analysis of *SERPINB3, -B4* and *MMP9* expression in A431 cells expressing shRNA against MCPIP1. *n = 3/5.* (**d**) qRT-PCR analysis of *SERPINB3/B4* and *MMP9* expression in A431 cells overexpressing MCPIP1 and treated with TPA for 24 h. *n = 3.* (**e**) Relative luciferase activity in HEK293 cells transfected with a luciferase reporter plasmid containing the 3’*-*UTRs of *SERPINB3/B4* and *MMP9* or the pmirGLO-empty vector together with the control plasmid or the expression plasmid expressing wild-type (WT) MCPIP1 or the D141N mutant. Luciferase activity was normalized to that of the pmirGLO-empty vector. *n = 4.* The data are shown as the mean ± SD values. Unpaired t-test (A-B) or one-way ANOVA (D-E) was used to calculate *P-values*. *P < 0.05, **P < 0.01, ***P < 0.001, ****P < 0.0001

Our RNA-seq analysis demonstrated overall elevated expression of serpin genes. Serpins are activated in response to elevated peptidase levels resulting from excessive inflammation. Human SERPIN B3 and B4 were discovered as tumor-specific antigens (and are thus referred to as SCC antigens SCCA1 and SCCA2, respectively) and are commonly used as tumor markers for SCC [32, 33]. Moreover, some isoforms (B3 and B10) have been reported to promote the survival of cancer cells by blocking proapoptotic signals [34, 35]. *Serpinb3a/b3b/b3c* and *-b3d* are orthologs of human SERPINB3 and B4 [36], and we confirmed their differential expression in Mcpip1^eKO^ papillomas (Fig. 5a). Another gene encoding an essential regulator of extracellular matrix metabolism whose level was significantly increased in Mcpip1^eKO^ tumors was *Mmp9* (Fig. 5a). Notably, its expression pattern was not altered in the untreated skin of aged mice [24], in contrast to the expression of Serpin B3 genes, whose expression was elevated in the whole skin of adult (3 months old) and aged (6-8 months old) Mcpip1^eKO^ mice not subjected to any treatment (Fig. 5b). We next developed A431 SCC cell lines with modulated expression levels of MCPIP1 *via* gene-specific shRNAs or overexpression of wild-type MCPIP1 or an MCPIP1 mutant with abolished RNase activity (D141N) (Fig. S2a and S2b, Additional file 4). Similar to the findings in chemically induced tumors in mice, the expression of the *SERPINB3, -B4* and *MMP9* genes was significantly increased in MCPIP1-silenced human malignant keratinocytes (Fig. 5c). Consistent with this finding, the levels of *SERPINB3, -B4* and *MMP9* were decreased in TPA-stimulated cells with ectopic expression of MCPIP1 (Fig. 5d). MCPIP1 is an RNase that functions through recognition of specific stem-loop structures [9]. We generated reporter constructs harboring the full-length 3’UTRs of human *SERPINB3/B4* (sharing 96% similarity in the 3’UTR) and *MMP9*. Luciferase assays showed that the 3’UTR of *MMP9* but not the 3’UTRs of *SERPINB3/B4* is most likely a direct target of MCPIP1, as indicated by the significant decrease in luciferase activity after expression of wild-type MCPIP1 but not the D141N mutant (Fig. 5e and S2c, Additional file 4).

### Elevated expression of protumorigenic factors in Mcpip1^eKO^ papillomas

**Fig. 6:**
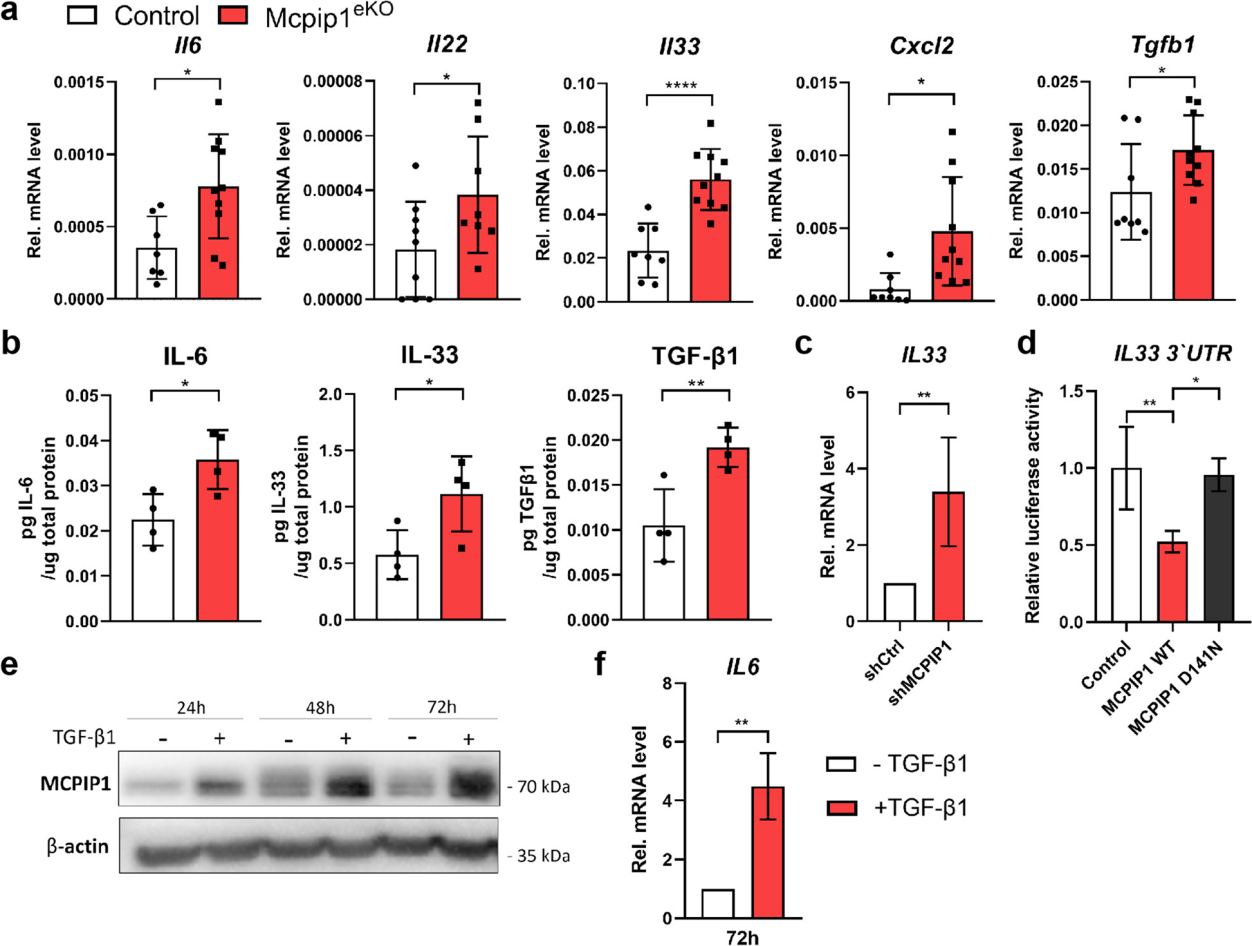
MCPIP1 modulates the expression of factors related to skin carcinogenesis. (**a-b**) RNA and protein were isolated from chemically induced papillomas. (**a**) qRT-PCR analysis of *Il6, Il22, Il33, Cxcl2* and *Tgfb1* mRNA expression. *n = 8-10*. (**b**) ELISA for IL-6, IL-33 and TGF-β1. *n = 4.* (**c**) qRT-PCR analysis of *IL33* mRNA expression in A431 cells stably expressing shRNAs. *n = 5.* (**d**) Relative luciferase activity in HEK293 cells transfected with luciferase reporter plasmids together with the control plasmid or plasmids encoding wild-type (WT) or mutated (D141N) MCPIP1. *n = 4.* (**e**) Representative western blot analysis of MCPIP1 protein expression in A431 cells stimulated with TGFβ1. β-Actin was used as the loading control. (**f**) qRT-PCR analysis of *IL6* mRNA expression in A431 cells stimulated with TGFβ1. *n = 3.* The data are shown as the mean ± SD values. Unpaired t-test (**a, b, c, e, f**) or one-way ANOVA (**d**) was used to calculate *P-values*. *P < 0.05, **P < 0.01, ***P < 0.001, ****P < 0.0001

To gain a deeper understanding of the inflammatory phenotype in Mcpip1^eKO^ tumors, we performed additional qRT-PCR and immunostaining analyses and found that the expression of *Il6, Ill22, Il33* and *Cxcl2* (cytokines/chemokines) and *Tgfb1* (a growth factor) was significantly enhanced in Mcpip1^eKO^ tumors (Fig. 6a). In contrast, the expression of the antigens *Cd19*, *GzmB*, *Cd14, Cd68*, and *Mrc1* (markers of B lymphocytes, NK cells, myeloid cells, macrophages and M2 macrophages, respectively) and Th2 cytokines (*Il4, Il5, Il10* and *Il13)* was unchanged, whereas the expression of the dermal dendritic cell antigen *Mgl2* and T lymphocyte antigen *Cd3e* was significantly decreased (Fig. S3a, Additional file 5). Immunostaining revealed that the numbers of F4/80-, CD68- and CD206-positive cells did not obviously differ between control and Mcpip1^eKO^ papillomas, consistent with the transcriptomic analysis results (Fig. S3b, Additional file 5). The differential expression of selected factors was also validated at the protein level by ELISA of whole tissue lysates. The papillomas in Mcpip1^eKO^ mice were characterized by increased protein levels of IL-6, IL-33 and TGF-β1 (Fig. 6b). Further, the mRNA level of IL-33 was increased in A431 cells with MCPIP1 silencing (Fig. 6c); the mechanism underlying this regulation involved direct binding of the MCPIP1 RNase to the *IL33* 3’UTR (Fig. 6d). Our studies suggested the involvement of TGF-β1 signaling in the development of the SCC-like phenotype in Mcpip1^eKO^ papillomas. We hypothesized that TGF-β1 is involved in a feedback loop to modulate MCPIP1 activity in malignant (or premalignant) keratinocytes. Indeed, stimulation of A431 cells with recombinant TGF-β1 led to increased protein expression of MCPIP1 (Fig. 6e). Interestingly, elevated expression of MCPIP1 did not lead to downregulation of its target, *IL6*, implying that the RNase becomes inactive upon stimulation with TGF-β1 (Fig. 6f).

## Discussion

Skin carcinogenesis is a complex, multistage process. Understanding its molecular basis is essential for its prevention and therapy. AK is a condition associated with cumulative sun exposure and is considered a common precursor of SCC. For a patient with AK, there are three possible outcomes: spontaneous clearance, persistence or progression to fully developed SCC [37]. Approximately 60-65% of diagnosed SCCs are believed to originate from AK lesions [3]; thus, early diagnosis of such abnormalities is a prerequisite to prevent malignant transformation. Diagnostic specificity and sensitivity may be improved by the development of specific molecular biomarkers. Here, we propose that MCPIP1 may become a novel biomarker for AK. We also speculate that its activity may be required to prevent or at least delay malignant transformation, as we found an obvious decrease in its expression in fully developed SCC lesions. However, currently, the exact mechanism of this regulation remains elusive.

In cancer, the expression and distribution of keratins is often dysregulated, and keratins are extensively used as tumor biomarkers [38]. Loss of KRT1 and KRT10, which are expressed in the suprabasal layers of the normal epidermis, is an indicator of an early stage of malignant conversion. Consistent with this observation, AK lesions showed a relatively normal distribution of KRT10 in the suprabasal epidermis, whereas KRT10 expression was reduced in fully developed SCC lesions. In contrast, the expression of KRT14, which was restricted to the narrow band of basal keratinocytes in both adjacent and AK tissues, was markedly elevated in malignant SCC lesions. In cancerous tissues, MCPIP1 expression was almost exclusively present in KRT10-expressing cells and was detected only occasionally in KRT14-positive cells, indicating that MCPIP1 expression is lost in cells with high proliferative potential. Additionally, in our animal model of SCC, gross changes in the KRT14/KRT10 staining pattern were observed. Compared to those in control mice, papillomas in Mcpip1^eKO^ mice were in a highly proliferative and dedifferentiated state. The increased expression of KRT14, accompanied by reduced expression of KRT10, in the Mcpip1^eKO^ papillomas indicated the acquisition of a more aggressive phenotype. Moreover, we observed a transcriptional increase in *Krt13* expression, which is characteristic of the progression of mouse papillomas into SCC [39, 40]. Generally, in early SCC tumorigenesis, exophytic papillomas are formed, which flatten over time [31]. The vast majority of Mcpip1^eKO^ papillomas showed endophytic growth accompanied by many morphological signs of early malignant transformation. In addition, angiogenesis was accelerated. Tumor angiogenesis is a complex process involving degradation of the extracellular matrix, cell migration and cell proliferation and is orchestrated by a plethora of cytokines/chemokines, growth factors, proteolytic enzymes and other mediators [41]. Among growth factors, VEGF, FGF and TGF-β are the most commonly associated with tumor angiogenesis [41, 42]. Moreover, inflammation is often described as the main inducer of angiogenesis during tumor growth, with IL-6 and IL-8 playing pivotal roles [41]. In the tumors formed in the skin of Mcpip1^eKO^ mice, enhanced expression of a plethora of proangiogenic factors (particularly IL-6 and TGF-β, among many others) was observed. To the best of our knowledge, this is the first *in vivo* study that associates the loss of MCPIP1 function with the promotion of tumor angiogenesis. Previous studies using mice were conducted with xenograft models [19]. As described above, tumor development is associated with elevated expression of proinflammatory genes, which was observed in Mcpip1^eKO^ mice. However, no obvious differences in the infiltration of major immune cell populations (except the T cell population) were noted, suggesting that in Mcpip1^eKO^ papillomas at 12 weeks post DMBA/TPA challenge, keratinocytes are the main source of the abovementioned proinflammatory factors. However, the influx of inflammatory cells may occur at later stages of tumor development. Interestingly, the T cell population was significantly decreased in Mcpip1^eKO^ tumors. High-risk papillomas have been shown to exhibit reduced T cell infiltration, suggesting the role of adaptive immunity in malignant conversion [43].

In this study, we found that serpin-encoding genes, orthologs of SCCA1/2, were highly expressed in the tumors formed in the skin of Mcpip1^eKO^ mice challenged with DMBA/TPA, suggesting progression from a benign to a premalignant or an advanced tumor phenotype. SCCA2-like serpins may confer genetic susceptibility to skin cancer in humans and in mouse models. In particular, high expression of SCCA2 was correlated with early onset of cutaneous SCC. In mice, injection of SCC cells overexpressing SCCA2 was found to accelerate tumor growth [44]. Here, we found that the expression of the orthologs SCCA1/SCCA2 was upregulated at the basal level in the skin of adult Mcpip1^eKO^ mice not subjected to any protumorigenic stimulus. However, Mcpip1^eKO^ mice do not spontaneously develop skin tumors but do develop skin inflammation upon aging, as we reported previously. In lesional skin, we noted the presence of keratin pearls, which are a typical characteristic of SCC [24]. We think that the presence of keratin pearls may be correlated with increased expression of SCC antigens. The exact mechanism by which MCPIP1 contributes to the regulation of serpins remains unknown. In keratinocytes, the expression of the orthologs SCCA1/2 is inversely correlated with the activity of MCPIP1; however, we verified that SCCA1/2 are not the direct targets of MCPIP1. One potential mechanism may involve the IL-17/IL-22 pathway, as the expression of SCCA molecules is induced by IL-22 in keratinocytes [32]. We previously reported accelerated activation of the IL-17/IL-23 pathway in the skin of Mcpip1^eKO^ mice challenged with imiquimod [28].

Our study showed that in malignant keratinocytes, MCPIP1 negatively regulates the levels of the MMP-9 and IL-33 transcripts by directly binding to their 3’UTRs. The metalloproteinase MMP-9 is involved in remodeling of the extracellular matrix to mediate tumor cell migration and invasion. In our model, a low level of MCPIP1 expression upregulated MMP-9. The expression of MMP-9 is also induced by TGF-β, a pleiotropic cytokine associated with many protumorigenic events [45]. A recent study by Taniguchi and coworkers described IL-33/TGF-β feedback signaling as a mechanism promoting the malignant progression of SCC [46]. Given that *IL33* is targeted by MCPIP1, one mechanism enhancing the expression of TGF-β in the epidermis expressing inactive MCPIP1 may involve upregulation of IL-33. Moreover, our preliminary results indicate that TGF-β acts on MCPIP1 *via* a feedback mechanism. This feedback leads to the stabilization of the inactive MCPIP1 protein, leading to upregulation of target mRNAs, such as the mRNA encoding IL-6. This mechanism most likely involves posttranslational modifications (a focus of our current investigation). Consistent with our results, TGF-β was previously demonstrated to stimulate the transcription of IL-6 in human retinal epithelial cells and human fibroblasts [47, 48]. Given our findings, we think that the mechanism underlying this regulation could be at least partially dependent on alterations in MCPIP1 activity.

Overall, we identified several factors that were upregulated in Mcpip1^eKO^ tumors, consistent with the observed protumorigenic phenotype. Based on our *in vivo* and *in vitro* studies, we speculate that in our model, the crosstalk between epithelial cells and the tumor environment (particularly immune cells) arises mainly *via* classical paracrine signaling (release of cytokines, chemokines, growth factors and other mediators) initiated by MCPIP1-deficient keratinocytes. It should be emphasized that Mcpip1^eKO^ mice do not develop skin tumors spontaneously but have substantially increased sensitivity to chemical carcinogenesis, which may actually arise from the persistent inflammatory environment. We previously demonstrated that Mcpip1^eKO^ mice develop signs of both local and systemic inflammation, which is a consequence of the continuous release of numerous proinflammatory factors from the skin. Chronic inflammation, when unresolved, may facilitate tumorigenesis [49, 50]. Therefore, MCPIP1 is emerging as an important player in skin tumorigenesis and may become a novel target of anticancer therapies.

## Conclusions

In summary, the level of MCPIP1 varies in the skin depending on the type of skin lesion, ranging from high in benign lesions such as AK to very low in advanced stages of malignant transformation such as SCC. Our studies in mice with keratinocyte-specific knockout of the gene encoding MCPIP1 and subjected to chemical carcinogenesis showed for the first time that the RNase MCPIP1 participates in tumor initiation and progression.

## Supplementary Information

**Additional file 1: Table S1.** List of primer sequences used for QRT-PCR in this study.

**Additional file 2: Table S2.** List of antibodies used in this study.

**Additional file 3: Figure S1.**

**Additional file 4: Figure S2.**

**Additional file 5: Figure S3.**

## Data Availability

Sequencing data are available in the European Nucleotide Archive under accession number PRJEB45882.

## Abbreviations

α-Sma: Alpha-smooth muscle actin
Casp14: Caspase 14
Chil1: Chitinase-like 1
Epgn: Epigen
Ereg: Epiregulin
FGF: Fibroblast growth factor
Hpse: Heparanase
KRT1: Keratin 1
KRT10: Keratin 10
KRT13: Keratin 13
KRT14: Keratin 14
Lif: Leukemia inhibitory factor
Spp1: Secreted phosphoprotein 1
TGF-β: Transforming growth factor beta
Tnfaip2: Tumor necrosis factor alpha-induced protein 2
Vegfa: Vascular endothelial growth factor a
