# Supplementary material for: Loss of epidermal MCPIP1 is associated with aggressive squamous cell carcinoma
Source: J Exp Clin Cancer Res. 2021 Dec 13;40:391. doi: 10.1186/s13046-021-02202-3 (PMC8667402; doi:10.1186/s13046-021-02202-3)
Supplement: Supplementary file 1 — Additional file 1: Table S1. List of primer sequences used for QRT-PCR in this study. [file 13046_2021_2202_MOESM1_ESM.docx]

**Additional file 1**

**Table S1.** List of primer sequences used for QRT-PCR in this study.

| **Gene Symbol** | **NCBI Reference** | **Forward** | **Reverse** | **Amplicon size [bp]** |
| --- | --- | --- | --- | --- |
| **Mouse** |  |  |  |  |
| *Ef2* | [NM_007907.2](https://www.ncbi.nlm.nih.gov/entrez/viewer.fcgi?db=nucleotide&id=237858599) | GACATCACCAAGGGTGTGCAG | TCAGCACACTGGCATAGAGGC | 214 |
| *Krt10* | [NM_010660.2](https://www.ncbi.nlm.nih.gov/entrez/viewer.fcgi?db=nucleotide&id=112983635) | GCTGGCCCTGAAACAATCGC | CAGGCTGCGGTAGGTCTGAA | 211 |
| *Krt14* | NM_016958.2 | TCCTGCTGGATGTGAAGACAAG | GCACATCCATGACCTTGGTGC | 186 |
| *Krt5* | [NM_027011.3](https://www.ncbi.nlm.nih.gov/blast/Blast.cgi?QUERY=NM_027011.3&PAGE=Nucleotides&PROGRAM=blastn&MEGABLAST=on&BLAST_PROGRAMS=megaBlast&PAGE_TYPE=BlastSearch&SHOW_DEFAULTS=on) | TGGACCAGTCAACATCTCTGTC | AGCTCATGCCTCCTTGACTG | 224 |
| *Krt13* | [NM_010662.2](https://www.ncbi.nlm.nih.gov/blast/Blast.cgi?QUERY=NM_010662.2&PAGE=Nucleotides&PROGRAM=blastn&MEGABLAST=on&BLAST_PROGRAMS=megaBlast&PAGE_TYPE=BlastSearch&SHOW_DEFAULTS=on) | CTCCGAAGTGAGATGGAGTGC | ACCCGTTGGAGGTAGTAGTG | 166 |
| *Vegfa* | NM_001025250 | CTTGTTCAGAGCGGAGAAAGC | ACATCTGCAAGTACGTTCGTT | 125 |
| *Lif* | NM_008501.2 | TCCTATTACACAGCTCAAGG | ATGGAAAGATGGGAAGTCTG | 87 |
| *Il1f9* | [NM_153511.3](https://www.ncbi.nlm.nih.gov/entrez/viewer.fcgi?db=nucleotide&id=224994268) | AGTTCCACGAAGCCACAGAGTA | TAGCAGCAAAGTAGGGTGTCCA | 166 |
| *Il20* | [NM_001311091.1](https://www.ncbi.nlm.nih.gov/projects/sviewer/sequence.cgi?netcache=0&id=gi\|902763187&format=fasta&filename=NM_001311091.1.fa&ranges=0-964) | TGAGAAAGATCAGCAGCCTCG | ATGCCTAGTTCTCCCAAAGCC | 184 |
| *Spp1* | [NM_001204201.1](https://www.ncbi.nlm.nih.gov/projects/sviewer/sequence.cgi?netcache=0&id=gi\|323668332&format=fasta&filename=NM_001204201.1.fa&ranges=0-1474) | GCACCCAGATCCTATAGCCAC | TGTGGTCATGGCTTTCATTGG | 149 |
| *Chil1* | [NM_007695.3](https://www.ncbi.nlm.nih.gov/projects/sviewer/sequence.cgi?netcache=0&id=gi\|224809460&format=fasta&filename=NM_007695.3.fa&ranges=0-1703) | TTACCAGACGCCATCCAACC | ATAAGAACGCAGGAACGGGG | 270 |
| *Ereg* | [NM_007950.2](https://www.ncbi.nlm.nih.gov/projects/sviewer/sequence.cgi?netcache=0&id=gi\|119709826&format=fasta&filename=NM_007950.2.fa&ranges=0-4135) | TGACGCTGCTTTGTCTAGGTT | ACACGGGGATCGTCTTCCAT | 130 |
| *Serpinb10* | [NM_198028.3](https://www.ncbi.nlm.nih.gov/projects/sviewer/sequence.cgi?netcache=0&id=gi\|237757329&format=fasta&filename=NM_198028.3.fa&ranges=0-3489) | ACGTACTTTGGGGCAGAACC | GCCTTTCGGTGGTGCTTTTC | 223 |
| *Klk12* | [NM_027097.1](https://www.ncbi.nlm.nih.gov/projects/sviewer/sequence.cgi?netcache=0&id=gi\|198278576&format=fasta&filename=NM_027097.1.fa&ranges=0-929) | GTCATGTCTCAGGATGGGGT | CAACAGATCCCCAGGAAACCA | 240 |
| *Klk13* | [NM_001039042.2](https://www.ncbi.nlm.nih.gov/projects/sviewer/sequence.cgi?netcache=0&id=gi\|110681721&format=fasta&filename=NM_001039042.2.fa&ranges=0-1032) | TTGGCCTTGTCCGAAGGAAT | TGCCCAGATGAACTGTGTACC | 229 |
| *Klk14* | [NM_174866.3](https://www.ncbi.nlm.nih.gov/projects/sviewer/sequence.cgi?netcache=0&id=gi\|914615964&format=fasta&filename=NM_174866.3.fa&ranges=0-1280) | ATTGTGCCCGCCCGATTC | GATACTGGGGATGTGGCACC | 111 |
| *Serpinb3a* | [NM_009126.3](https://www.ncbi.nlm.nih.gov/projects/sviewer/sequence.cgi?netcache=0&id=gi\|226958517&format=fasta&filename=NM_009126.3.fa&ranges=0-1628) | ATTCCTGGGTAGAAAGCCAAA | GCACATCCTCCAGAAACATGA | 240 |
| *Serpinb3b* | [NM_198680.2](https://www.ncbi.nlm.nih.gov/projects/sviewer/sequence.cgi?netcache=0&id=gi\|142354324&format=fasta&filename=NM_198680.2.fa&ranges=0-1631) | ATCCTTCCTGCTAACTTCTGCC | TCAGCTGCATGAAAACGAATCA | 83 |
| *Serpinb3c* | [NM_201363.2](https://www.ncbi.nlm.nih.gov/projects/sviewer/sequence.cgi?netcache=0&id=gi\|160333612&format=fasta&filename=NM_201363.2.fa&ranges=0-1658) | ACAGGCTTCTATCCTTCCTGCT | CCATCAGCTTCAGGAAAAAGAATCA | 96 |
| *Serpinb3d* | [NM_201376.1](https://www.ncbi.nlm.nih.gov/projects/sviewer/sequence.cgi?netcache=0&id=gi\|41235786&format=fasta&filename=NM_201376.1.fa&ranges=0-1163) | CCCCATCAGCATGATGAGAAC | ACTGTTGGGAACCTTTAGGTCA | 223 |
| *Mmp9* | NM_013599.4 | CTCTGCTGCCCCTTACCAG | AGCGGTACAAGTATGCCTCTGC | 107 |
| *Il6* | NM_031168.2 | ACTTCACAAGTCGGAGGCTT | GGTACTCCAGAAGACCAGAGG | 220 |
| *Il22* | NM_016971.2 | AGACAGGTTCCAGCCCTACA | CCAGTTCCCCAATCGCCTT | 179 |
| *Il33* | NM_001360725.1 | AGGCGACGGTGTGGATGGGA | CGTCACCCCTTTGAAGCTCCACG | 116 |
| *Cxcl2* | NM_009140.2 | CCCAGACAGAAGTCATAGCCAC | CGAGGCACATCAGGTACGAT | 221 |
| *Tgfb1* | NM_000358.3 | AGACCTTGAACCGCATCCTG | AGATGACAGCCTTCCCGTTG | 186 |
| *Cd19* | NM_001357091.1 | GCCACAGCTTTAGATGAAGGCAC | CATCCACCAGTTCTCAACAGCC | 116 |
| *GzmB* | [NM_013542.3](https://www.ncbi.nlm.nih.gov/entrez/viewer.fcgi?db=nucleotide&id=1068937234) | CTGCTCACTGTGAAGGAAGTATAA | AGCTCTAGTCCTCTTGGCCT | 185 |
| *Cd14* | [NM_009841.4](https://www.ncbi.nlm.nih.gov/entrez/viewer.fcgi?db=nucleotide&id=669033312) | CTCTGTCCTTAAAGCGGCTTAC | GTTGCGGAGGTTCAAGATGTT | 191 |
| *Mrc1* | NM_008625.2 | CGTGGATTCCTTTCTATGGC | ACACAATCATTCCGTTCACCA | 289 |
| *Mgl2* | XM_017314459.1 | AGGCACCCTAAGAGCCATTT | CCCTCTTCTCCAGTGTGCTC | 200 |
| *Cd3e* | [NM_007648.5](https://www.ncbi.nlm.nih.gov/projects/sviewer/sequence.cgi?netcache=0&id=gi\|285026447&format=fasta&filename=NM_145822.2.fa&ranges=0-2247) | GCTCCAGGATTTCTCGGAAGTC | ATGGCTACTGCTGTCAGGTCCA | 138 |
| *Il4* | [NM_021283.2](https://www.ncbi.nlm.nih.gov/entrez/viewer.fcgi?db=nucleotide&id=226874825) | TCGGCATTTTGAACGAGGTC | GAAAAGCCCGAAAGAGTCTC | 216 |
| *Il5* | [NM_010558.1](https://www.ncbi.nlm.nih.gov/entrez/viewer.fcgi?db=nucleotide&id=6754335) | AGCACAGTGGTGAAAGAGACCTT | TCCAATGCATAGCTGGTGATTT | 117 |
| *Il10* | NM_010548.2 | ACAACATACTGCTAACCGACT | AGAAATCGATGACAGCGCCTC | 205 |
| *Il13* | NM_008355.3 | GCCGGTGCCAAGATCTGTGTC | CTCCATACCATGCTGCCGTTG | 111 |
| **Human** |  |  |  |  |
| *SERPINB3* | NM_006919.3 | GCTGAAGATCGCCAACAAGC | CCAATGTGGTATTGCTGCCA | 224 |
| *SERPINB4* | NM_002974.4 | GCTGAAGATCGCCAACAAGC | GCGTTCACAAGAACCAGTGTC | 237 |
| *MMP9* | NM_004994 | CGCAGACATCGTCATCCAGT | GGATTGGCCTTGGAAGATGA | 406 |
| *IL33* | NM_033439.4 | AACACCCCTCAAATGAATCAGGT | GAGTGTTCCTTGTTGTTGGCA | 106 |
| *IL6* | NM_000600 | GTGAAAGCAGCAAAGAGGCA | TCACCAGGCAAGTCTCCTCA | 108 |
| **Cloning** |  |  |  |  |
| *SERPINB3/B4 3`UTR* | NM_006919.3NM_002974.4 | ATGCTAGCGGTTTTTAGACACATTGC | ATGTCGACGCAAATGAGATAAATG | 460 |
| *MMP9 3`UTR* | NM_004994 | ATGCTAGCTACGTGACCTATGACAT | ATGTCGACTAAAGGTTAGAGAATCCAAGTTTATTAG | 234 |
| *IL33 3`UTR* | NM_033439.4 | ATGCTAGCGGTTTTTAGACACATTGC | ATGTCGACGCAAATGAGATAAATG | 1815 |
