# Supplementary material for: Loss of epidermal MCPIP1 is associated with aggressive squamous cell carcinoma
Source: J Exp Clin Cancer Res. 2021 Dec 13;40:391. doi: 10.1186/s13046-021-02202-3 (PMC8667402; doi:10.1186/s13046-021-02202-3)
Supplement: Supplementary file 2 — Additional file 2: Table S2. List of antibodies used in this study. [file 13046_2021_2202_MOESM2_ESM.docx]

**Additional file 2**

**Table S2.** List of antibodies used in this study.

| **Antigen** | **Host species** | **Working dilution (method)** | **Catalogue number** | **Company** |
| --- | --- | --- | --- | --- |
| **Primary antibodies** |  |  |  |  |
| PCNA | Mouse | 1:200 (IF) | M0879 | Dako Cytomation, Hamburg, Germany |
| Keratin 14 | Mouse | 1:200 (IF) | ab7800 | Abcam, Cambridge, UK |
| Keratin 10 | Mouse | 1:200 (IF) | ab9026 | Abcam, Cambridge, UK |
| Keratin 10 | Rabbit | 1:200 (IF) | ab76318 | Abcam, Cambridge, UK |
| CD31 | Rat | 1:100 (IF) | 550274 | BD Pharmingen™, San Jose, CA, USA |
| αSMA | Rabbit | 1:100 (IF) | ab5694 | Abcam, Cambridge, UK |
| CD68 | Rabbit | 1:200 (IF) | ab125212 | Abcam, Cambridge, UK |
| CD206 | Rat | 1:200 (IF) | MCA2235GA | BioRad, California, USA |
| MCPIP1 | Rabbit | 1:2000 (WB) | - | Own production |
| MCPIP1 | Rabbit | 1:200 (IHC) | GTX110807 | GeneTex, Inc., CA, USA |
| β-actin | Mouse | 1:2000 (WB) | A1978 | Sigma Aldrich, Saint Louis, MO, USA |
| **Secondary antibodies** |  |  |  |  |
| peroxidase-conjugated anti-rabbit | Goat | 1:20 000 (WB) | A0545 | Sigma Aldrich, Saint Louis, MO, USA |
| peroxidase-conjugated anti-mouse | Goat | 1:20 000 (WB) | 554002 | BD Pharmingen™, San Jose, CA, USA |
| AF488 anti-mouse | Goat | 1:600 (IF) | A11001 | Invitrogen, Darmstadt, Germany |
| AF488 anti-rabbit | Goat | 1:600 (IF) | A11008 | Invitrogen, Darmstadt, Germany |
| AF546 anti-rabbit | Goat | 1:600 (IF) | A11035 | Invitrogen, Darmstadt, Germany |
| AF546 anti-mouse | Goat | 1:600 (IF) | A11003 | Invitrogen, Darmstadt, Germany |
| AF594 anti-rat | Goat | 1:600 (IF) | A11007 | Invitrogen, Darmstadt, Germany |
