# Supplementary material for: Loss of epidermal MCPIP1 is associated with aggressive squamous cell carcinoma
Source: J Exp Clin Cancer Res. 2021 Dec 13;40:391. doi: 10.1186/s13046-021-02202-3 (PMC8667402; doi:10.1186/s13046-021-02202-3)
Supplement: Supplementary file 3 — Additional file 3: Figure S1. [file 13046_2021_2202_MOESM3_ESM.docx]

**Additional file 3 - Figure S1**

**
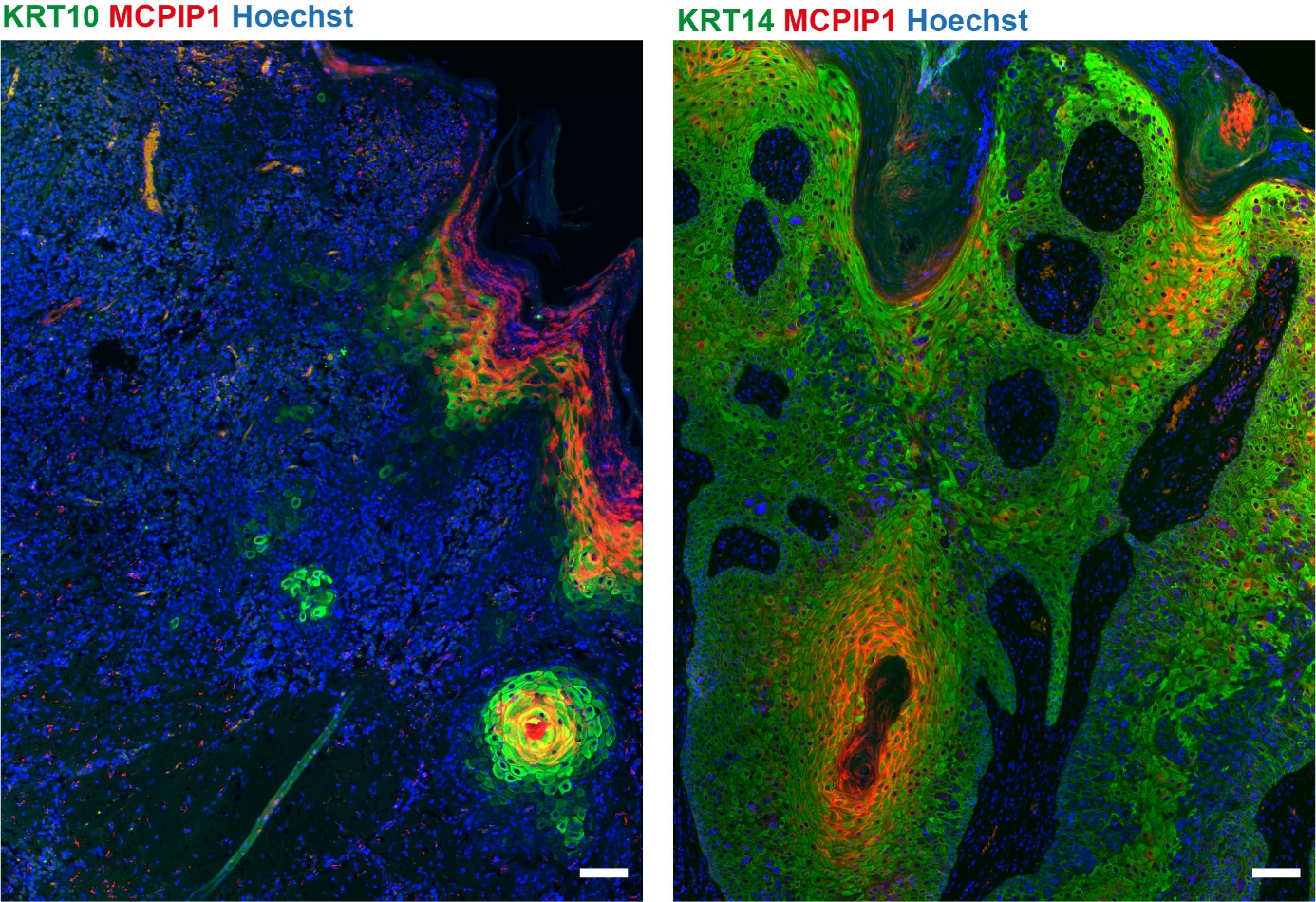
**

**Figure S1.** Keratin 10 (KRT10), Keratin 14 (KRT14) and MCPIP1 immunofluorescence staining of human squamous cell carcinoma skin biopsies. Scale bar: 100 μm.
