# Supplementary material for: Loss of epidermal MCPIP1 is associated with aggressive squamous cell carcinoma
Source: J Exp Clin Cancer Res. 2021 Dec 13;40:391. doi: 10.1186/s13046-021-02202-3 (PMC8667402; doi:10.1186/s13046-021-02202-3)
Supplement: Supplementary file 4 — Additional file 4: Figure S2. [file 13046_2021_2202_MOESM4_ESM.docx]

**Additonal file 4 - Figure S2**

**
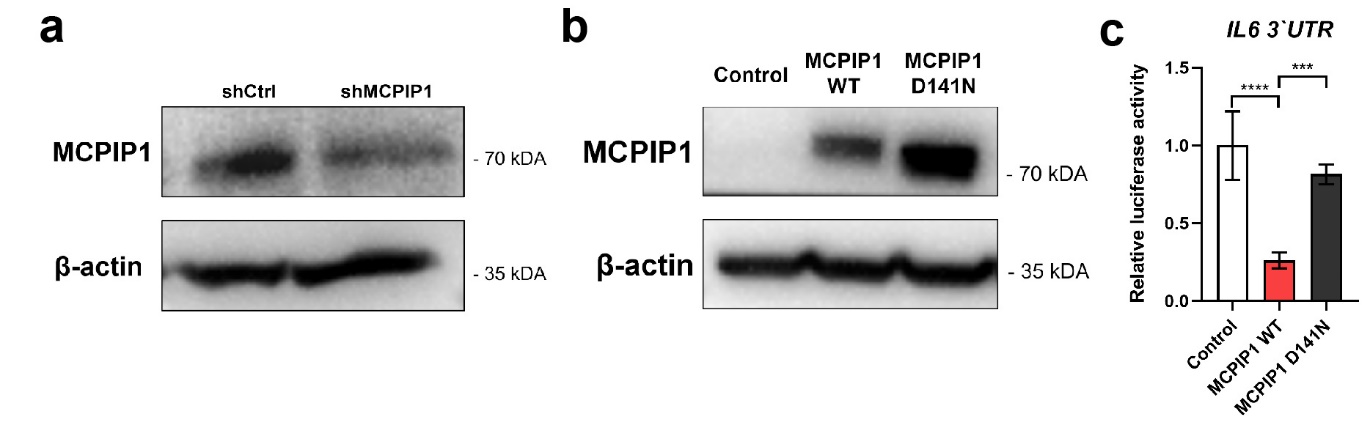
**

**Figure S2. (a)** Representative western blot analysis for MCPIP1 and β-actin in A431 cells stably expressing control shRNA or shRNA against MCPIP1. **(b)** Representative western blot analysis for MCPIP1 and β-actin in A431 cells overexpressing wild type or mutated MCPIP1 (D141N) and treated with 5ng/ml TPA for 24 hours. **(c)** Relative luciferase activity of HEK293 cells co-transfected with luciferase reporter plasmid (pmirGLO) containing *3`-*UTR sequences of *IL6* gene **[1]**, together with control or an expression plasmid encoding wild-time MCPIP1 (MCPIP1 WT) or mutant with abolished RNase activity (MCPIP1 D141N). Luciferase activity was normalized to the pmirGLO-empty vector. *n = 4*. Data are shown as a mean ± SD. One-way ANOVA was used to calculate *P-values*. ****P* < 0.001, *****P* < 0.0001.

1. Kochan J, Wawro M, Kasza A. Simultaneous detection of mRNA and protein in single cells using immunofluorescence-combined single-molecule RNA FISH. Biotechniques. 2015 Oct;59(4):209–12. doi: 10.2144/000114340.
