# Supplementary material for: Loss of epidermal MCPIP1 is associated with aggressive squamous cell carcinoma
Source: J Exp Clin Cancer Res. 2021 Dec 13;40:391. doi: 10.1186/s13046-021-02202-3 (PMC8667402; doi:10.1186/s13046-021-02202-3)
Supplement: Supplementary file 5 — Additional file 5: Figure S3. [file 13046_2021_2202_MOESM5_ESM.docx]

**Additional file 5 – Figure S3**

**
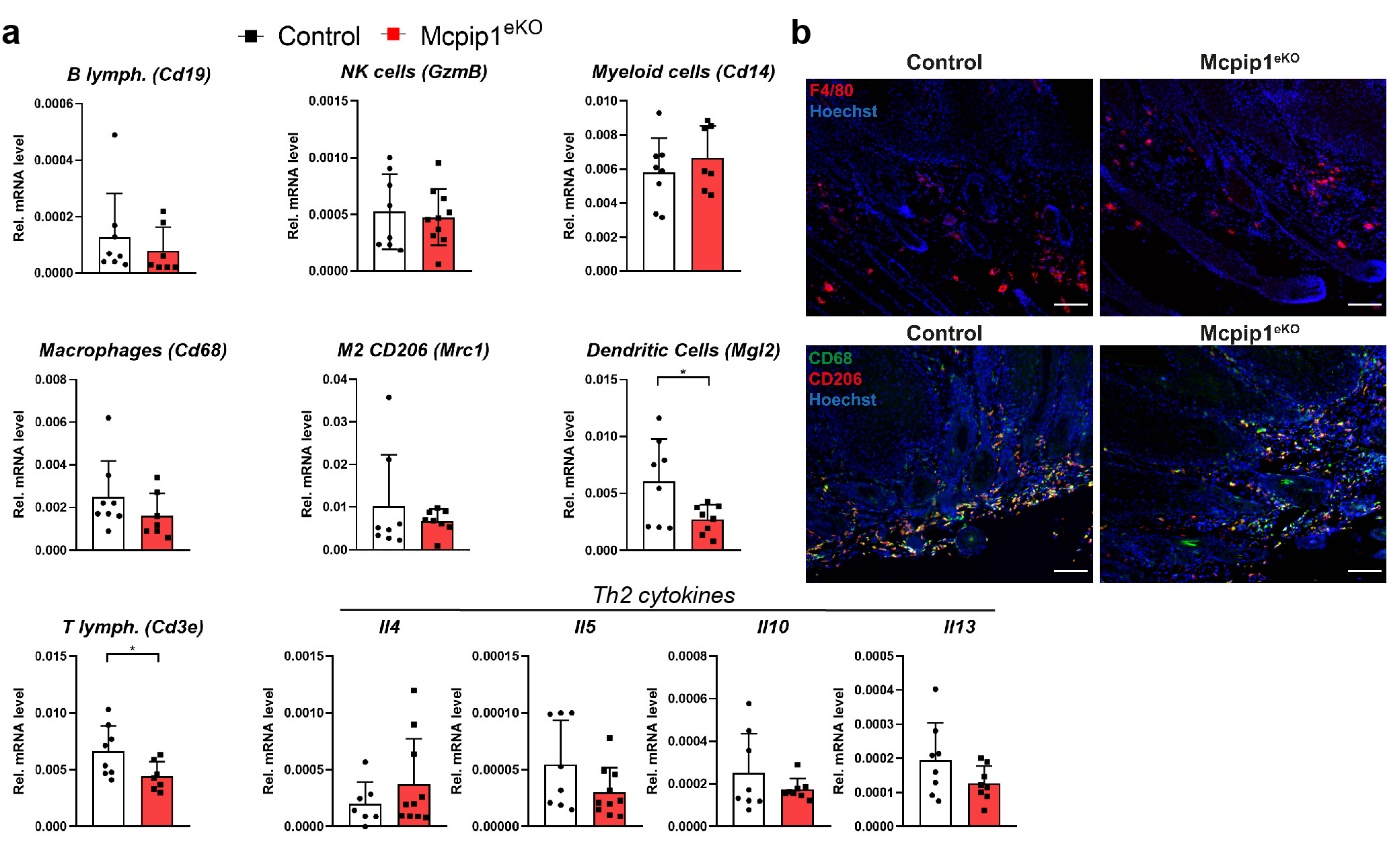
**

**Figure S3. (a)** qRT-PCR analysis of *Cd19, GzmB, Cd14, Cd68, Mrc1, Mgl2, Cd3e, Il4, Il5, Il10* and *Il13*. *n = 8.* Data are shown as a mean ± SD. Unpaired t-test was used to calculate *P-values*. **P* < 0.05. **(b)** F4/80 and CD68/CD206 immunofluorescence in Mcpip1^eKO^ and control papillomas. Scale bar: 100 μm.
